# Assessment of RV systolic function by tissue motion annular displacement in healthy cats

**DOI:** 10.1007/s11259-026-11201-8

**Published:** 2026-04-17

**Authors:** Edwin F. Buriticá, Giovana L. R. Tuleski, Vinícius B. C. Silva, Joelma F. Santos, Marlos G. Sousa

**Affiliations:** 1 Laboratory of Comparative Cardiology, Department of Veterinary Medicine, Federal University of Paraná (UFPR), Rua dos Funcionarios 1540, Curitiba, 80035-050 Brazil; 2 Small Animal Medicine and Surgery Research Group, Faculty of Veterinary Medicine and Zootechny, University of Tolima (UT), Santa Helena Cll42 1- 02, Ibagué, 730006299 Colombia

**Keywords:** Echocardiography, Systole, Right ventricle, TAPSE, TMAD

## Abstract

Tissue motion annular displacement (TMAD) of the tricuspid valve, obtained by speckle-tracking echocardiography, evaluates RV longitudinal systolic function (RVLSF) by measuring annular displacement toward the ventricular apex. This study aimed to assess right ventricular (RV) function in 56 clinically healthy cats using TMAD and to compare the results with conventional echocardiographic parameters and longitudinal strain of the RV free wall (LSt-fw). Mean TMAD values were 4.8 ± 1.1 mm, 23.5 ± 3.8%, 17.9 ± 5.2 mm/m², and 1.1 ± 0.4 mm/kg. TMAD measured in millimeters and percentage showed positive correlations with tricuspid annular plane systolic excursion (TAPSE) (*r* = 0.23, *p* = 0.034; and *r* = 0.37, *p* = 0.005, respectively). Agreement analysis demonstrated moderate repeatability, with biases of -0.62 (intraobserver) and − 0.34 (interobserver) for TMAD in millimeters, and − 0.36 and − 1.36, respectively, for TMAD in percentage. In addition, the time required to obtain TMAD measurements was significantly shorter than that for LSt-fw (median 10.5 s vs. 28.0 s; *p* < 0.0001). These findings indicate that TMAD is a feasible and rapid method for evaluating RVLSF in healthy cats.

**Supplementary Information:**

The online version contains supplementary material available at 10.1007/s11259-026-11201-8.

## Introduction

Right ventricular longitudinal systolic function (RVLSF) is a key prognostic indicator in the progression of both congenital and acquired cardiac diseases, including atrial and ventricular septal defects (Oliveira et al. 2011), pulmonary hypertension (Morita et al. 2019) and arrhythmias (Meurs 2017). Echocardiographic assessment of RVLSF is complex due to the anatomical characteristics of the right ventricle (Mertens and Friedberg 2010; Wessels et al. 2021). Traditionally, several echocardiographic indices such as tricuspid annular plane systolic excursion (TAPSE), fractional area change (FAC), and peak velocity of tricuspid annular motion measured by pulsed wave Doppler (S’) have been widely used in clinical practice to estimate RVLSF (Visser et al. 2015). However, these parameters have important limitations, including angle dependency, load sensitivity, and reliance on geometric assumptions (Ahmad et al. 2012). To overcome these constraints, speckle-tracking echocardiography has emerged as an advanced technique for evaluating RVLSF mechanics through myocardial deformation indices, providing advantages such as angle independence and improved sensitivity to subtle functional changes (Silva et al. 2020). Moreover, reference values with good repeatability and potential clinical applicability have been proposed for dogs (Wolf et al. 2018; Silva et al. 2020).

Tissue motion annular displacement (TMAD) uses speckle-tracking echocardiography to quantify annular displacement toward the ventricular apex during systole, assuming relative apical stability throughout the cardiac cycle (Choi et al. 2013). Good correlation and satisfactory reproducibility have been reported between TMAD applied to the mitral annulus and left ventricular global longitudinal strain in healthy dogs (Wolf et al. 2018) and cats (Tuleski et al. 2022). However, TMAD has not yet been evaluated for right ventricular performance in cats; therefore, this study investigated its feasibility for assessing RV longitudinal systolic function in healthy cats.

## Animals, materials and methods

This prospective, cross-sectional observational study enrolled 56 client-owned cats of different breeds and ages. Healthy cats admitted for elective procedures at a Veterinary Teaching Facility or specifically recruited were evaluated. All cats underwent a complete physical examination, systolic blood pressure (SBP) measurement, and electrocardiographic and echocardiographic assessments. Cats with abnormalities in the parameters mentioned above, as well as those with sonographic evidence of acquired or congenital heart disease, were excluded. All procedures were approved by the institutional Ethics Committee on Animal Use (protocol 014/2019).

### Echocardiography

Echocardiographic examinations were performed using a 4.0–12.0 MHz phased-array transducer (Affiniti, Koninklijke Philips N.V., Amsterdam, The Netherlands). Cats were without sedation and positioned in left and right lateral recumbency in accordance with American College of Veterinary Internal Medicine guidelines (Thomas et al. 1993). All studies were conducted by a single operator (G.L.R.T.), and all measurements were obtained by a single investigator (V.B.C.S.).

### Conventional echocardiographic parameters

For conventional assessment of RV morphology, two-dimensional systolic and diastolic measurements (in millimeters) included the basal internal diameter (measured from the free wall to the interventricular septum immediately below the tricuspid annulus), the mid-ventricular diameter at the medial third, and RV length (measured from the apex to the tricuspid annular plane). All echocardiographic measurements were performed with simultaneous ECG recording.

The RVLSF was assessed using FAC, recognized as the percent difference between systolic and diastolic RV area; TAPSE, measured between the most basilar position of the tricuspid annulus in end diastole and its most apical displacement at end systole; and tissue Doppler S’ at the same junction. Only one measurement was obtained for linear parameters (structural measures, TAPSE, and FAC).

### Parameters derived from speckle tracking

Speckle-tracking analysis was performed using the QLAB software with the Automated Cardiac Motion Quantification (aCMQ) application (Affiniti, Koninklijke Philips N.V., Amsterdam, The Netherlands). For speckle-tracking analysis, two-dimensional images were acquired from an apical four-chamber view optimized for visualization of the right ventricle. All images used for speckle-tracking analysis were acquired at a frame rate of at least 100 frames per second. LSt-fw was automatically derived after defining the region of interest (ROI) using three fixed points (RV septal annulus, RV lateral annulus, and RV apex). Although strain was calculated for all segments, only the three free-wall segments (basal, mid, apical) were used within a single cardiac cycle. For tricuspid annular motion displacement (TMAD), three ROIs were selected: lateral (P1) and septal (P2) tricuspid annuli and the epicardial RV apex; tracking was performed automatically. TMAD was calculated as displacement of the virtual midpoint between the two annular ROIs toward the apex (MP, mm) and as a percentage relative to total RV length (MP, %). TMAD was also indexed to body weight (BW) (mm/kg) and body surface area (mm/m²), calculated as BSA = 0.100 × (BW^^0.67^). One investigator measured the time needed to obtain the TMAD value.

### Intraobserver and interobserver variability and analysis time

Repeatability was assessed in 16 cats. Intraobserver variability was evaluated by repeat measurements performed by the same observer after ≥ 30 days, and interobserver variability was determined by a blinded second investigator (G.L.R.T.) using the same datasets. Offline analysis time for LSt-fw and TMAD was recorded for these cats. These 16 cats were not randomly chosen but represented a convenience sample from the study population.

### Statistic analysis

Statistical analysis included Shapiro–Wilk testing for normality. Parametric data were reported as mean ± SD with minimum–maximum, while nonparametric data were described using median, interquartile range, and minimum–maximum. Group differences were evaluated with an unpaired t-test (parametric) or Mann–Whitney U test (nonparametric). Correlations between TMAD and the structural or functional variables to assess RV function were assessed using Pearson (parametric) or Spearman (nonparametric) tests. Intra- and interobserver agreement were assessed using the intraclass correlation coefficient (ICC) with 95% CI and standard error of measurement (SEM). Analyses were conducted with SPSS 21.0 and GraphPad Prism 9.0, considering *p* < 0.05 as significant.

## Results

A total of 56 cats were enrolled in the study, including Persian (*n* = 3), Maine Coon (*n* = 2), Sphynx (*n* = 1), and predominantly mixed-breed cats (*n* = 50). Of these, 30 were female (54%) and 26 were male (46%). The mean age was 5.3 ± 1.5 years, and the mean BW was 4.1 ± 1.0 kg.

Table 1 presents descriptive statistics for clinical and echocardiographic variables in the cats studied. TMAD (mm), TMAD (%), and P1 (mm) showed significant correlations with TAPSE; however, no correlations were observed with other systolic function variables, except for TMAD (mm), which also correlated with S′. TMAD (mm), P1 (mm), and P2 (mm) correlated with S′ and RV length in diastole. TMAD (mm) correlated with most structural parameters, except for mean RV diameter in diastole, whereas P1 (mm) correlated with most structural variables except for RV length in systole. No significant correlations were identified between indexed TMAD values (mm/m² and mm/kg) and any echocardiographic variables. No correlations were found between TMAD (mm) or TMAD (%) and age, HR, SBP, BW or sex.

**Fig. 1:**
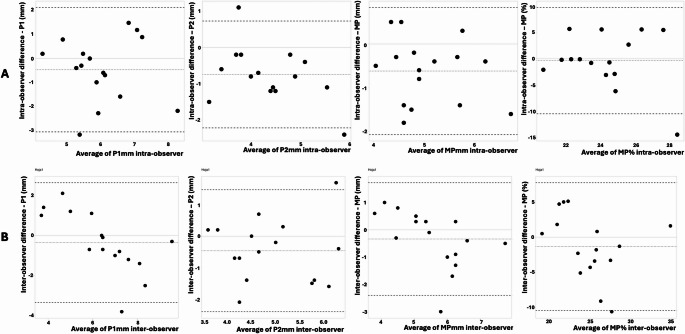
Bland-Altman plots showing intra- and inter-observer agreement for TMAD measurements. Panel **A** displays intra-observer agreement, while Panel **B** shows inter-observer agreement. The solid line indicates the mean bias, and the dashed lines mark the 95% limits of agreement

The inter and intra-observer variations are displayed in Table 2 for all the TMAD indexes. The intraobserver agreement for MPmm analysis revealed a bias of − 0.62 (LOA, − 2.07 to 0.83) and for interobserver of − 0.34 (LOA, − 2.40 to 1.72). For intraobserver of MP% agreement analysis exposed a bias of − 0.36 (LOA, − 10.52 to 9.80) and for interobserver of − 1.36 (LOA, − 10.45 to 7.73). Figure 1 displays the Bland-Altman graphics for TMAD (mm) and TMAD (%) variables and their 95% limits of agreement.

The mean time to complete TMAD was shorter compared with LSt-fw (10.5, IQR 9.4–11.3 and 28.0 ± 10.4 s, respectively), with statistical significance (*p* < 0.0001).

**Table 1:** Descriptive statistics (minimum, maximum, mean or median, and standard deviation or interquartile range) for age, weight, systolic blood pressure, heart rate, and echocardiography variables for the cats studied

Variables	*n*	Min	Max	Mean or median	SD or IQR
Age (Years)	55	1,0	13,0	5,4	3,4
Weight (Kg)	55	2,1	11,6	4,7	1,5
SBP (mmHg)	54	100,0	159,0	140,0	130–150
HR (bpm)	55	140,0	265,0	196,2	28,4
P1 (mm)	55	2,7	8,4	5,5	1,4
P2 (mm)	55	2,4	7,3	4,1	3,6 − 4,7
TMAD - MP (mm)	56	2,5	7,5	4,8	1,1
TMAD - MP (%)	56	15,7	30,6	23,5	3,8
TMAD - MP (mm/m^2^)	55	7,2	34,4	17,9	5,2
TMAD - MP (mm/Kg)	55	0,3	2,3	1,1	0,4
LSt fw	56	17,7	43,8	27,9	5,5
TAPSE	56	4,4	13,1	8,7	1,8
FAC (%)	55	39,0	78,8	59,9	10,5
S’	46	6,6	21,3	10,5	9,3–12,3

SBP: systolic blood pressure; HR: heart rate; TMAD: tissue motion annular displacement; MP: displacement of a virtual midpoint between the two tricuspid valve ROI and the right ventricle apex; MP%: percentage of the midpoint relative to the total length of the right ventricle; P1mm: value of the displacement of the lateral tricuspid annulus; P2mm: value of the displacement of the septal annulus; MP mm/m^2^: value of the midpoint in relation to the total length of the right ventricle, expressed in mm/m^2^; MP mm/kg: value of the midpoint relative to the total length of the right ventricle, expressed in mm/kg; LSt fw: longitudinal strain of the right ventricle free wall; TAPSE: tricuspid annular plane systolic excursion; FAC %: fractional area change percentage; S’: systolic myocardial velocity of the lateral tricuspid annulus obtained from pulsed wave tissue Doppler imaging

**Table 2:** Intra and inter-observer variations for tissue motion annular displacement (TMAD) variables using the apical four-chamber image

Variable	ICC (0–1)	SEM	M	SD	95%CI
Intraobserver variation					
T MAD P1 (mm)	0,49	0,30	6,28	1,22	5,63–6,93
T MAD P2 (mm)	0,47	0,25	4,68	1,01	4,14–5,22
T MAD (mm)	0,47	0,22	5,38	0,88	4,91–5,85
T MAD (%)	0,40	0,93	24,35	3,70	22,38 − 26,32
Interobserver variation					
T MAD P1 (mm)	0,64	0,55	6,64	2,22	5,46–7,82
T MAD P2 (mm)	0,48	0,25	5,14	1,01	4,60–5,68
T MAD (mm)	0,58	0,35	5,72	1,38	4,98–6,46
T MAD (%)	0,45	1,26	25,71	5,05	23,01 − 28,40

## Discussion

This study aimed to evaluate the feasibility, repeatability, and analysis time of TMAD for assessing right ventricular longitudinal systolic function in healthy cats and found that this method is quick, reproducible, and primarily correlates with TAPSE and S′.

Knowledge of RVLSF is clinically important in cats because right ventricular remodeling and dysfunction have been observed in feline hypertrophic cardiomyopathy and in cats with pulmonary hypertension secondary to left-sided congestive heart failure (Schober et al. 2016). However, because of anatomical and physiological differences from the left ventricle, RV assessment is more complex and often requires complementary techniques (Mertens and Friedberg 2010). This is the first study to describe right heart function in cats using TMAD.

The RV contraction is primarily driven by longitudinal fiber shortening from the base toward the apex (Rushmer et al. 1953) and by inward motion of the free wall toward the interventricular septum (Wessels et al. 2021). TAPSE measures the systolic longitudinal movement of the tricuspid annulus toward the RV apex. Its main limitation is its one-dimensional approach and the need for precise beam alignment, which can lead to underestimating displacement (Ahmad et al. 2012). In humans, TMAD can be obtained from a single apical four-chamber view and is less affected by suboptimal endocardial definition, dropout, and insonation angle (Terada et al. 2022), suggesting that it may be less influenced than TAPSE.

In dogs, TMAD shows moderate correlations with several indices of ventricular systolic function, including TAPSE, FAC, and speckle-tracking derived parameters (Wolf et al. 2018); however, only one study has specifically evaluated these associations in the right heart (Silva et al. 2020). In the present study, TMAD correlated only with TAPSE and S′, which may be explained by the fact that all three variables primarily reflect longitudinal base-to-apex motion of the tricuspid annulus. The lack of correlation with other echocardiographic variables of RV function may be related to species-specific differences in cardiac size and anatomical position within the thorax, as well as to the inherent technical difficulty of RV assessment in cats. In this context, TMAD should be interpreted as a complementary index of RV longitudinal systolic function rather than as a standalone surrogate of overall RV systolic performance. In addition, P1mm measured at the lateral tricuspid annulus was higher than P2mm measured at the septal annulus. This finding may be related to two factors: (1) TAPSE may be influenced by left ventricular function due to ventricular interdependence (Li et al. 2015), and (2) the lateral RV wall is more directly associated with inflow mechanics, whereas the septal region is influenced by both ventricles.

Mean TMAD values in this study were higher than those reported for the left ventricle in cats (Tuleski et al. 2022; Glaewketgarn and Surachetpong 2025) but lower than those described for the right ventricle in dogs (Silva et al. 2020). These differences may be partly explained by variation in sampling-point location among studies. Myocardial deformation is heterogeneous, varying not only between ventricular walls but also within the same wall, from base to apex and from subendocardial to subepicardial layers (Chetboul et al. 2004). In the present study, all speckle-tracking points were placed in the subendocardial region. Moreover, the RV is predominantly composed of longitudinally oriented fibers and exhibits minimal short-axis deformation, whereas the left ventricle is characterized mainly by radial and circumferential fibers (Spotnitz 2000; Spalla et al. 2019). This structural difference may explain why indices of longitudinal systolic function are typically higher in the RV than in the left ventricle.

Echocardiographic assessment of RV morphology is challenging due to its small size, proximity to the near field of the ultrasound beam, and the presence of strong reflectors, such as the pericardial sac and epicardial fat, which may lead to overestimation of wall thickness. In addition, the RV’s complex geometry and prominent endocardial trabeculations further hinder accurate wall thickness measurements (Schober et al. 2016). In dogs, TMAD has been shown to correlate positively with BW (Wolf et al. 2018; Silva et al. 2020); however, this relationship appears less consistent in cats. In the present study, TMAD did not correlate with BW, in agreement with Tuleski et al. (2022), who suggested that adult cats have more homogeneous cardiac dimensions than dogs.

The mean time required to obtain TMAD measurements in this study was 10.5 s (IQR, 9.4–11.3 s), which was significantly shorter than that required for RV free-wall longitudinal strain (LSt-fw) analysis (28.0 ± 10.4 s). This difference is likely attributable to the greater operator-dependent effort needed to identify, track, and correct speckle patterns along the RV wall when measuring LSt-fw, particularly in the presence of ultrasound artifacts that commonly affect RV imaging. In contrast, TMAD relies on fewer tracking points at the annular level, facilitating faster acquisition and analysis.

Notably, both intra- and interobserver analyses demonstrated acceptable correlation and agreement for TMAD measured in millimeters and as a percentage, indicating moderate repeatability. These findings suggest that TMAD is a rapid and reproducible method for assessing RV systolic function in cats, consistent with results reported in previous studies in other species (Ahmad et al. 2012; Wolf et al. 2018). Bland–Altman analysis showed mean differences close to zero, suggesting no relevant systematic bias, but the relatively wide limits of agreement indicate moderate variability between repeated measurements. Clinically, we believe this variability is unlikely to affect interpretation when TMAD values are clearly normal or clearly abnormal, but it may influence judgment in borderline cases. Therefore, TMAD should be interpreted cautiously and in conjunction with other indices of RV systolic function.

This study has several limitations. Cats were classified as healthy based on physical examination, echocardiography, electrocardiography, and blood pressure measurements; however, the presence of subclinical or asymptomatic comorbidities cannot be completely excluded. Additionally, using a blood pressure cutoff below 160 mmHg does not completely exclude cats with mildly elevated blood pressure, even though they have a low risk of target organ damage. Furthermore, the sample size and limited breed diversity may not be fully representative of feline populations in other geographic regions. S′ data were available only in a subset of cats because this measurement was not consistently acquired or stored in all examinations, which should be considered when interpreting results related to this variable. The TMAD software used in this study has not been specifically validated for RV assessment. Furthermore, echocardiography may be less sensitive than cardiac magnetic resonance imaging for detecting subtle structural or functional abnormalities, particularly in the right ventricle, whose complex geometry makes accurate assessment more challenging.

## Conclusions

TMAD appears to be a promising complementary tool for assessing RV longitudinal systolic function in healthy cats, alongside conventional echocardiographic methods. It is rapid to perform, demonstrates acceptable repeatability, is not influenced by BW in cats, and shows correlation with TAPSE and longitudinal measurements of the RV free wall. However, further studies are warranted to determine the clinical applicability of this technique in cats with cardiac and non-cardiac diseases.

## Supplementary Information

Below is the link to the electronic supplementary material.

Supplementary Material 1

## Data Availability

The datasets generated during and/or analysed during the current study are available from the corresponding author on reasonable request.

## Abbreviations

BSA: Body surface area
BW: Body weight
FAC: Fractional area change
FW: Free wall
HR: Heart rate
ICC: Intraclass correlation coefficient
LSt-fw: Longitudinal strain of the RV free wall
ROI: Regions of interest
RV: Right ventricular
RVLSF: Right ventricular longitudinal systolic function
S’: Peak velocity of systolic tricuspid annular motion as determined by pulsed wave Doppler
SBP: Systolic blood pressure
SEM: Standard error of measurement
TAPSE: Tricuspid annular plane systolic excursion
TMAD: Tissue motion annular displacement
